# An Asian Body to Tackle Cancers in Asia – The Asian National Cancer Centers Alliance

**DOI:** 10.31557/APJCP.2020.21.5.1207

**Published:** 2020-05

**Authors:** Laureline Gatellier, Tomohiro Matsuda, Kanaga Sabapathy, Min Dai, Luh Komang Mela Dewi, Tran Thanh Huong, Kardinah Kardinah, Tran Van Thuan, Jong Bae Park, Jie He, Erdenekhuu Nansalmaa, Bayarsaikhan Luvsandorj, William Ying Khee Hwang, Manju Sengar, CS Pramesh, Tatsuya Suzuki

**Affiliations:** 1 *National Cancer Center, Japan, 5-1-1 Tsukiji, Chuo-ku, Tokyo 104-0045, Japan. *; 2 *National Cancer Centre Singapore, 11, Hospital Crescent, Singapore.*; 3 *National Cancer Center, China, 17 Panjiayuan Nanli, Chaoyang District, Beijing 100021, China. *; 4 *Dharmais Hospital - National Cancer Center, Jalan Letjend S. Parman No.84-89 Kecamatan Palmerah, Kota Jakarta Barat, DKI Jakarta, 11420, Indonesia. *; 5 *National Cancer Institute & Hanoi Medical University, Hanoi, Vietnam. *; 6 *National Cancer Center of Korea, 323 Ilsan-ro, Ilsandong-gu, Goyang-si Gyeonggi-do, 10408, Republic of Korea. *; 7 *National Cancer Center of Mongolia, Nam Yan Ju Street, 13370 Ulaanbaatar, Mongolia. *; 8 *Tata Memorial Hospital, Dr. E, Dr Ernest Borges Rd, Parel, Mumbai, Maharashtra 400012, India *

**Keywords:** Cancer burden, Asia- cancer control, inequalities, survival

## Abstract

The socioeconomic burden of cancer is growing rapidly in the Asian region, with a concentrated burden on low- and middle- income countries. The residents of this region, representing almost 60% of the global population, demonstrate an eclectic and complex nature, with huge disparities in ethnicity, sociocultural practices among others. The Asian National Cancer Centers Alliance (ANCCA) was established in 2005 by heads of several national cancer centers (NCCs) in the region to address common issues and concerns among Asian countries. During the first 13 years of ANCCA’s existence, the participating NCCs’ senior managers paved the way toward collaboration through transparent sharing of key facts and activities. Concrete achievements of the Alliance include the Asia Tobacco-Free Declaration, the establishment of the ANCCA Constitution in 2014 as well as the creation of an official website more recently. In November 2019, the most active ANCCA members (China, India, Indonesia, Japan, Korea, Mongolia, Singapore, Thailand, and Vietnam) strengthened the bonds of the entity with the clear aim to halt the increase in cancer and mortality rates in Asian countries by 2030. New opportunities including accelerated cooperation between members as well as collaboration with external and multidisciplinary stakeholders at local, regional and international levels are an essential step to most effectively tackle cancers in Asia.

## Introduction

Over the past five decades, Asian countries have achieved remarkable economic success, with the Asia – Pacific region contributing to more than two-thirds of the global economic growth (IMF, 2018; IMF, 2019). Contrary to the current high economical expectations in the region, the cancer toll in Asia remains a growing “global health threat”, where cancer is responsible for 57.3% of the world population cancer deaths while the population in Asia is 59.5% of that of the globe (Bray et al., 2018). Even though the cancer incidence rate is lower in Asia compared to the West, the mortality to incidence ratio is higher (Ng et al., 2015) and the cancer burden is expected to increase due to various reasons: aging, growing populations, and striking variations in ethnicity, sociocultural practices, socioeconomic status, and human development index among others (Sankaranarayanan et al., 2014; Grover, 2015). 

In addition, as Asia is an eclectic continent due to all inter-country differences as well as “Asian geographical specificities” (Singh and Teo, 2019), the anticipated benefits of collaboration among countries are greater than ever. Any change in Asia, which counts currently for 60% of the global population, will have a global impact. Given the wide disparity of HDI index, low-middle countries are more in urgent need to set a National Cancer Control Plan (Arnold et al., 2016), and focus on domestic prevention, screening and medical care, while High HDI index Asian countries, are more in the position to support their neighbors, and share their experience and knowledge to build a stronger Asian continent. Collaboration would further promote research and improve care of cancers more prevalent in the region. 

Several forms of collaboration have been put in place in cancer prevention, control, cancer registries, Universal Health Coverage (UHC) with support from and collaboration with the International Agency for Research on Cancer (IARC), International Association of Cancer Registries (IACR), the Asian Pacific Organization for Cancer Prevention (APOCP), the Association of Southeast Asian Nations (ASEAN) and UICC-Asia Regional Office (UICC-ARO), currently leading the way to strengthen collaboration on the Asian continent in the fight against cancer (Woodward, 2014) (Akaza et al., 2017). Collaboration among Asian national cancer centers appointed by the Ministry of Health has been sporadically established through official bilateral agreements such as memoranda of understanding with neighboring Asian countries or with other institutions or agreements on specific projects, but until the Asian National Cancer Centers Alliance (ANCCA) was established, a multilateral framework for regional collaboration was non-existent.

**Background of ANCCA**

*History of ANCCA*

ANCCA was officially launched at the first meeting in September 2005 where the heads of 9 national cancer centers from Asia (Bangladesh, China, Japan, Korea, Mongolia, Pakistan, Singapore, Thailand and Vietnam) started the initiative to embark together on a fight against cancer and to collaborate as an alliance with the World Health Organization (WHO) and IARC. A landmark ANCCA agreement was further formalized in 2007, through a bylaw agreed by 10 NCC Heads who gathered for their second meeting in Tokyo, Japan, in which NCC India also joined. The Head of NCC Singapore was appointed as the first Secretariat-General of ANCCA. The purpose of ANCCA in 2007 was to address the growing cancer threat to the people living in Asia by promoting collaboration among members to advance cancer prevention, cancer research, clinical trials, training and plans or guidelines. As agreed in the bylaw, rotating bi-annual ANCCA meetings, including center tours, allowed all Heads to discuss and share challenges and achievements in their fight against cancer in Asian countries. Governmental advocacy was also initiated through a common Asia Tobacco-Free Declaration agreed at the 5th ANCCA meeting in 2014 in Goyang, Korea, as encouragement to promote legislative measures aimed to ultimately ban tobacco in each ANCCA member country.

ANCCA’s framework was also reinforced by the ANCCA Constitution, where NCC Korea was officially endorsed as ANCCA secretariat, another step towards a more sustainable entity. During the first 13 years of its existence, ANCCA has successfully coordinated actions against cancer, such as the “Tobacco-Free Asia” Declaration in 2014, however, it still needs consistent efforts to reach long-term goals.

New initiatives included the invitation of new ANCCA members, such as India in 2007, Nepal and Turkey in 2009, and Malaysia in 2014. International organizations have also been invited to actively collaborate with ANCCA, with the participation to regular meetings of the International Prevention Research Institute (IPRI) (in 2009 and 2011), IARC, and later on (2014 and 2018), the Asia Pacific Organization for Cancer Prevention (APOCP). 

**Figure 1:**
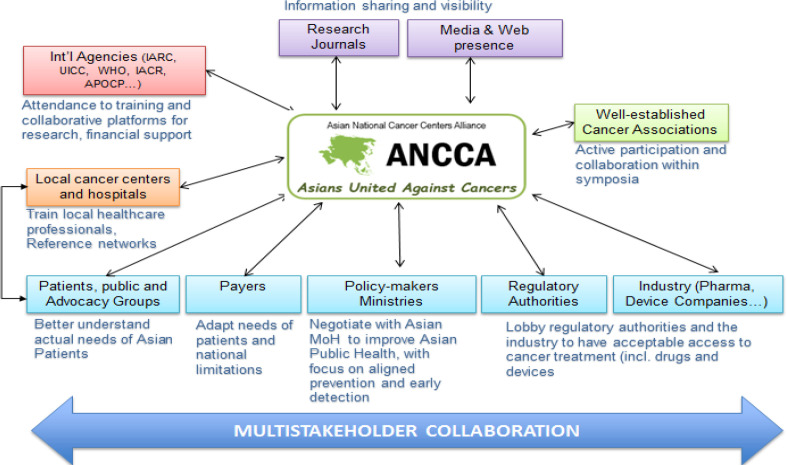
ANCCA as a Key Player in Asia, to Promote the Best Cancer Care for the Asian Continent

ANCCA collaboration reached a key milestone at the 7^th^ ANCCA meeting in October 2018 in Jakarta under the leadership of NCC Indonesia (Dharmais Hospital) where all members emphasized the need for collaborative actions among ANCCA members and with external stakeholders in an era where cancer management is at the forefront of natural health priorities. At the very minimum, members consider further collaboration with local cancer centers and hospitals, patients, public and advocacy groups, payers, policy makers, regulatory authorities, the industry and well established cancer associations important to promote superior cancer care for the Asian continent (Figure 1).

*National cancer centers (members) – Key facts and activities*

ANCCA members have a unique commonality through their appointment by the Ministry of Health of each country, in turn providing a leadership nationally. As a necessity to efficiently collaborate among NCCs, the first step is understanding each other’s strengths and weaknesses, from historical, geographical, political as well as economical perspective. An overview of key members who mostly contributed in 2018-19 is provided below in alphabetical order. 

*China*

NCC China (NCC-CN) was established in 2011 by Ministry of Health, China, based on the structure of Cancer Institute and Hospital, Chinese Academy of Medical Sciences (CIHCAMS) built in 1958. As a leading organization of cancer prevention and control in China, NCC-CN’s main mission is to prevent cancer, conquer cancer and serve for cancer patients. It’s main activities include 1) Draft the national cancer prevention and control plan (NCCP) and lead the nationwide cancer prevention and control programs; 2) Carry out the advanced diagnosis and treatment for cancer patients and draft the guidelines; 3) Train professionals in cancer prevention and treatment; 4) Carry out the basic research on cancer and promote the application and popularization of new knowledge and technology into the practice of cancer prevention and control.

*India*

Tata Memorial Hospital (NCC-IN) was initially commissioned by the Sir Dorabji Tata Trust in 1941, based on a humanitarian commitment. In 1962, the center was brought under the Government of India (Department of Atomic Energy) and later merged as Tata Memorial Centre (NCC-IN). The original mandate of Service, Education and Research in Cancer stands as true today as during its inception. NCC-IN is considered as the premier research center for cancers relevant to India and South-East Asia. In addition, NCC-IN also collaborates with other organizations such as IARC (epidemiology) or NIH, USA on largescale research studies. NCC-IN also leads one of its kind initiative National Cancer Grid, a collaboration of 203 cancer centers, charitable organizations and patient representatives, to ensure uniform cancer care, healthcare professional training and multi-centric research across India. 

*Indonesia*

NCC Indonesia (NCC-ID) was inaugurated in 1993 as Dharmais Hospital, the National Referral Hospital for Cancer in Indonesia. In 2017, Dharmais Hospital was established as National Cancer Center of Indonesia by the Ministry of Health and accountable to provide comprehensive cancer services, cancer research, oncology education and training, as well as to develop cancer registration in Indonesia. Dharmais National Cancer Center has national cancer registry network with 14 national referral hospitals and official partnership with cancer institution from 7 countries with main activities on early detection and screening of breast cancer, and education for oncology professionals. 

*Japan*

National Cancer Center Japan (NCC-JP) was established by the Ministry of Health and Welfare in 1962, commissioned to lead the nation’s cancer control strategies and policies. With over 3,500 staff, including 600 physicians, 1,200 nurses and 1,000 beds, the center, including 2 hospitals and 5 other main departments, is implementing latest technologies such as interventional radiology, proton-therapy and BNCT. The Center for Cancer Genomics and Advanced Therapeutics (C-CAT) was established in 2018, to provide high-quality cancer genomic medicine in Japan. NCC-JP also dedicates its resources in rare cancers, establishing the “Rare Cancer Center” in 2014 towards the clinical development of rare cancers in Japan (Kawai et al., 2018), and with the MASTER KEY Project, a platform (basket/umbrella) trial with a registry study for rare cancers toward faster new drug approvals (Okuma and Fujiwara, 2019). 

*Korea*

NCC Korea (NCC-KR) was established in 2000, following the National Cancer Center Act enactment. In 2005, the National Cancer Control Institute and Research building opened, followed in 2007 by the National Cancer Prevention and Detection building and in 2014, by the Graduate School of Cancer Science and Policy. In addition to education and training, NCC-KR is very active in research, patient care, and support for national cancer control programs. NCC-KR Hospital maintains a patient-oriented medical care system, by operating 16 multidisciplinary centers, including 11 organ-specific cancers. As cancer control programs, NCC-KR functions as the Headquarter for the population-based National Cancer Registry. Among others, the cancer Patients Management program, Financial Aid program and Code of conduct for Cancer Prevention show NCC-KR’s key dedication to cancer patients.

*Mongolia*

The National Cancer Centre of Mongolia was established as a state radiotherapy hospital in 1961 and renamed as the NCC-MN in 2006 with the vision to become a premier center for providing quality medical services and treatments for cancer patients. The NCC-MN is a sole state medical institution which delivers friendly, equitable and quality services nationwide. 

The NCC-MN ensures a leadership in implementing technological innovations and new knowledge in cancer prevention, cancer diagnosis and cancer treatment. NCC-MN has developed early cancer detection and cancer registry data collection systems as well as evidence based information, research and methodology in its activities and decisions. 

NCC-MN believes that introduction of early detection plan for cancer management will have a positive impact to the treatment protocol and will result in reducing cancer mortality and saving lives of many people.

*Singapore*

NCC Singapore (NCC-SG) is committed to being a global leading cancer center through research, education and clinical services and patient care, was officially opened in 1999, and became part of Singapore Health Services (SingHealth) in 2000. NCC-SG, which is Southeast Asia’s only full multi-disciplinary sub-specialist center for cancer, focuses on Asian Cancer Genomics, Clinical Trials, Cancer Therapeutics, Proton Beam Therapy, Cancer Genetics, Support and Palliative Care and Translational Imaging. As part of Patient Care, the Cancer Genetics Services is well established as a platform to support healthcare decisions involved in precision medicine. NCC-SG sees over 60% of cancer patients in Singapore and performed 8,524 day surgeries and received 156,170 outpatient visits in 2018.

*Thailand*

Thailand’s National Cancer Institute (NCC-TH) was established in 1968, under the leadership of His Majesty King Bhumibol Adulyadej of Thailand. NCC-TH’s goal is to be the nation’s foremost leading cancer institute by developing and improving knowledge and technology for cancer to positively impact cancer control and patient care. Examples of concrete focus in the four main pillars (“health policy”, “prevention and early detection”, “diagnostic, treatment and palliative care” and “surveillance and research”) are respectively the incorporation of regional cancer control issues, the reduction of carcinogen exposure, the development on new and the development of population-based registries. 

*Vietnam*

NCC Vietnam “K Hospital” (NCC-VN) was established in Hanoi in 1923 and originally named “Institut du Radium de l’Indochine”. This facility is the largest hospital specializing in oncology in Vietnam, and includes 4 campuses with more than 1800 beds. In 2007, the Vietnam Ministry of Health decided to establish the National Institute for Cancer Control (NICC) in the K hospital. The NICC is responsible for the development of research on cancer care, and coordinates the National Target Programs for Cancer Control. The NICC carries out a variety of research activities, such as clinical trials and population-based studies on breast cancer and colorectal cancer. It is accredited for offering resident and clinical fellow training programs in oncology, a Master of Science degree in Oncology, and a Ph.D. training program in oncology for both basic scientists and clinical oncologists. In 2019, NCC-VN counted 76 departments and centers, providing highest quality service of cancer diagnosis and treatment, allowing activities such as through targeted therapies with the establishment of the Clinical Research Center and the Laparoscopic and Robotic Surgery Center respectively in 2018 and 2019. Other activities include national and international research and collaboration in the field of cancer registry as well as knowledge-sharing.

**ANCCA Action Plans**

*Focus on the future collaboration in ANCCA activities*

ANCCA was created by 9 national cancers centers to unite and decrease the cancer burden through open collaboration among members. By learning from each other, members have identified national and regional priorities and confirming the importance to collaborating and supporting each other in fields such as medical training, education, prevention, and national cancer control plans. ANCCA activities have gained pace in 2018 with the establishment of a new ANCCA website (www.ancca.asia), for smoother collaboration and international visibility. 

**Figure 2:**
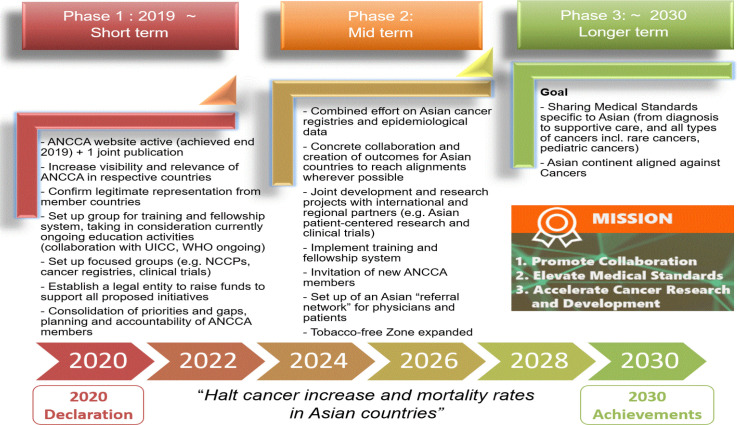
ANCCA Achievements, Short Term, Mid-Term and Longer-Term Goals

In the year following the 7^th^ ANCCA meeting, a total of 5 regular video conference have been conducted, under the leadership of NCC-KR and NCC-JP and the ANCCA website was launched by end 2019, through strong involvement of NCC Indonesia. Around the same time, all attending members agreed on ANCCA’s activity plans, from the short (several months to months), mid-term (one year to several years) and longer term vision (with the aim to “halt cancer increase and mortality rates in Asian countries” by 2030) (Figure 2 – ANCCA achievements, short term, midterm and long term goals)

Planning for and creating short-wins, such as education and training, or alignment on registries is initiated, while next key focus of ANCCA should be to institutionalize new approaches within a newly established framework. The collaborative projects that have already been initiated will in turn fall under the scope of this new framework. Future directions and longer-term project goals could include the potential initiation of an “Asia cancer plan” to include the minimum requirements for all Asian countries. 

*Phase 1 – 2020 to 2022*

Sustainability cannot be achieved without a focus on ANCCA visibility and clear commitments. With some short-term achievements and the launch of the ANCCA website, regular updates and access to key activities, key research, comparative registry data and patient services on the website are starting to allow active multistakeholder communication. 

In order to sustainably remain a key player in this war against cancers, the organization should aim from the start, to form a powerful guiding coalition, which would allow secure highly committed staff as well as financial consistency. Needed, and already started at this early stage is the evaluation of the long term strategy in parallel with the invitation of financial, legal, human resources commitment from all members (Knaul et al., 2015). Rather than a heavy resource-based entity, members appreciate ANCCA’s role in providing ground data and acting as a think-tank to clarify Asian common issues as national and regional specificities and needs are shared. As most members are leading cancer centers mandated by their respective ministries of health, concrete plans can be discussed closely with and endorsed by policy makers and regulators of each country (See Figure 1 – ANCCA Asia as a key player in Asia), anticipated to be, in turn, linked with national goals and grants, such as AMED grant in Japan. 

In this journey, several ANCCA members are leading specific paths from different perspectives such as: NCC Korea, Singapore and China investigating co-leadership regarding education and training. India has proposed to lead the cancer registries project, attractive to most members, as well as sharing the activities of the global component of the Indian National Cancer Grid Indonesia, in addition to its keen implication through the website establishment, is interested in most initiatives, starting with cancer registries and training. Japan and Mongolia are encouraged by other members to lead the NCCP and healthcare system comparison project, with several other members showing interest. The clinical trial project has attracted the interest of most members, with Japan as a potential coordinator. 

Not to be forgotten, and emphasized as a concrete project under Singapore’s leadership, communicating ANCCA’s activities and achievements through publications and active presence in scientific meetings would be beneficial in encouraging new partners to take part in activities in the Asian region and worldwide.

*Phase 2 – 2023 to 2027*

Considered as midterm activities, while ANCCA is reaching its stable and regular pace, involve financial, legal and administrative aspects. Embarking on this challenging journey, ANCCA members have started investigating ways to strengthen the entity through legal, financial means while setting strategic priorities, communicating to current and potential new national cancer centers based in Asia who would be interested to join as new members. Work is currently ongoing and may lead to constitution update. 

Another key project expected in Phase 2 is the participation in clinical trials, which is also an essential part of reducing the world-wide cancer burden, especially exciting in this era of genomic and precision medicine. Several members already play an active role in the approval of new drugs not only locally, but also support approval processes in other major regulatory frameworks. 

In addition to these new mid-term activities, projects planned at Phase 1 mentioned above are to be implemented, such as “tobacco free zone” expansion activities. Rare cancers, which represent around 20 % of all cancers in Asia as well in Europe (Matsuda et al., in press ; Gatta et al., 2011), is also a promising field for joint action, calling for alignment of Asian forces, which could eventually play a greater role in global improvement of cancer care.

*Phase 3 – 2028 to 2030*

Concrete achievements to be reached by 2030 by ANCCA in all fields started in Phase 1 and implemented in Phase 2 include among others Asian Continent Aligned Cancer prevention activities. Other expected improvements are Asian-specific medical standards (from diagnosis to supportive care, for all types of cancers, including rare and pediatric cancers), as a result of ANCCA collaboration in NCCPs, cancer registries, education and training, clinical trials and other joint research projects. One key goal of ANCCA members is to improve the quality of cancer care in order to fully comply with high international standards at each participating center.

## Discussion

In the new era of big data and AI and while genomic and immunologic advances are speeding up mainly under the leadership of north American and Europe (Car et al., 2019), ANCCA’s “Asians United Against Cancers” shows a new path where its competitive advantage is in the fact that it is led by Asians and for Asians with the clear purpose of swiftly achieving a reduction of the Asian cancer burden. Collaboration with well-established international organizations will leverage activities initiated elsewhere to boost collaboration of ANCCA members within the region. 

ANCCA’s competitive advantage is worth leveraging for the benefit of cancer patients not only in Asia but is noticeable also on a global scale. Its geographic position, economies of scale and scope could make ANCCA a key player through its competitive advantage as a “passion driven” group of academic / scientific institutions with governmental support. Such collaboration is ever more urgently called for as the economic burden of cancer is sky-rocketing, with ever more expensive drugs (Fitzner et al., 2017; Malik, 2009), making them unavailable to cancer patients in countries and regions although they may have participated in clinical trials for its development, but where the healthcare system cannot allow fair access to them due to the high cost (Ruiz et al., 2017).

This key role of ANCCA can be achieved through members’ current privileged working relationships with decision-makers and government, while future establishment of multi-stakeholder collaboration, also with payers, the general public and patients from the region (Pollock et al., 2018) would be key to improve cancer care in Asia. The future of ANCCA will depend on a well-established constitution, which will play a key role in securing appropriate funding and financial support for sustainability. As a key step to achieve such a position, more emphasis is currently given in securing real-world based evidence on survival data in the Asian region. 

In conclusion, ANCCA as the most prominent official group of leading cancer centers in Asia is already contributing to decreasing the cancer burden in Asia (see Figure 2 – ANCCA’s achievements and goals), and is expected to expand its contribution through key tools such as ongoing collaboration (education and training, visibility etc) with multiple stakeholders such as international organizations, governments, payers.
